# Preliminary investigation of elevated collagen and blood‐clotting markers as potential noninvasive biomarkers for small cell lung cancer

**DOI:** 10.1111/1759-7714.15066

**Published:** 2023-08-19

**Authors:** Jeppe Thorlacius‐Ussing, Søren Risom Kristensen, Morten Asser Karsdal, Nicholas Willumsen, Shona Pedersen

**Affiliations:** ^1^ Nordic Bioscience A/S Herlev Denmark; ^2^ Department of Clinical Medicine Aalborg University Aalborg Denmark; ^3^ Department of Clinical Biochemistry Aalborg University Hospital Aalborg Denmark; ^4^ Department of Basic Medical Sciences, College of Medicine, QU Health Qatar University Doha Qatar

**Keywords:** biomarkers, collagen, extracellular matrix, small cell lung cancer

## Abstract

**Background:**

Small cell lung cancer (SCLC) is highly aggressive with limited therapeutic options and a poor prognosis. Moreover, noninvasive biomarker tools for detecting disease and monitoring treatment response are lacking. To address this, we evaluated serum biomarkers of extracellular matrix proteins not previously explored in SCLC.

**Methods:**

We measured biomarkers in the serum of 16 patients with SCLC before and after chemotherapy as well as in the serum of 11 healthy individuals.

**Results:**

Our findings demonstrated that SCLC serum had higher levels of collagen type I degradation, collagen type III formation, and collagen type XI formation than healthy controls. In addition, we observed higher levels of type XIX and XXII collagens, fibrinogen, and von Willebrand factor A formation in SCLC serum. The formation of type I collagen did not exhibit any discernible variation. However, we observed a decrease in the degradation of type I collagen following chemotherapy.

**Conclusion:**

Overall, our findings revealed elevated levels of collagen and blood‐clotting markers in the serum of SCLC patients, indicating the potential of ECM proteins as noninvasive biomarkers for SCLC.

## Graphical abstract

The tumor microenvironment of small cell lung cancer (SCLC) accumulates extracellular matrix (ECM) proteins including collagens. Fragments of these proteins find their way to the circulation, where they can be detected in blood by immunoassays and may be used as cancer biomarkers. Created with BioRender.

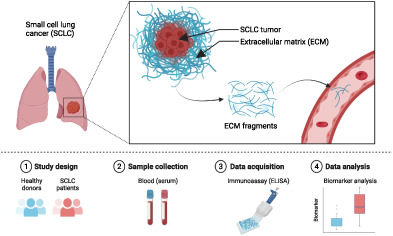

## INTRODUCTION

Small cell lung cancer (SCLC) is a rapidly progressive form of cancer with a poor prognosis that accounts for approximately 15% of all lung cancer cases.^1^ Despite recent advances, such as promising outcomes from immunotherapy,^2^ treatment options for SCLC are still restricted and primarily involve chemotherapy drugs such as platinum agents (cisplatin or carboplatin) and topoisomerase inhibitors (etoposide or irinotecan). Although the response rates following standard‐of‐care chemotherapy or radiation are initially high in SCLC, chemoresistance arises frequently and rapidly.^3^

While the mechanisms of chemoresistance have not yet been fully elucidated, recent research has shown that processes such as epithelial to mesenchymal transition (EMT) are now known to play a role^4^, ^5^ and fibroblasts, and extracellular matrix (ECM) proteins such as collagens, laminins, fibronectin, and proteoglycans,^6^ promote this transition.^7^, ^8^ Moreover, patients with SCLC often present with symptoms of advanced disease, including weight loss, fatigue, and poor wound healing,^1^ with an increased risk of thromboembolism.^9^ Although the mechanisms are not fully understood, the abnormal production of procoagulant factors, such as tissue factor, von Willebrand factor, or fibrinogen, may be involved,^9^ and thromboembolism continues to be a significant cause of cancer‐related mortality.^9^

Identifying and validating reliable biomarkers for SCLC has proven difficult, partly because of the heterogeneity of the disease and partly because of the scarcity of tissue samples.^10^ Nonetheless, developing noninvasive biomarker methods, such as liquid biopsies, does not rely on tissue sampling and may broaden the use of biomarkers in SCLC diagnosis and management. Over the past decade, Willumsen et al. have shown the potential of ECM biomarkers as liquid biopsies in the context of non‐small cell lung cancer (NSCLC), and several of these may also show potential in SCLC.^11^, ^12^, ^13^, ^14^, ^15^, ^16^ Interestingly, in SCLC, alterations in the expression and composition of ECM proteins have been associated with chemoresistance,^17^, ^18^ and collagen expression is increased in lung cancer, which is thought to contribute to tumor growth and invasion.^19^, ^20^, ^21^ In addition, degradation has also been associated with a more aggressive phenotype.^22^ Furthermore, ECM interacts with cell surface receptors, such as integrins, that activate signaling pathways to regulate cancer cell behavior, including chemoresistance.^22^

In this preliminary investigation, we explored the potential of ECM and wound healing biomarkers in the serum of 16 patients with SCLC before and after chemotherapy as well as 11 healthy controls with similar age and gender proportions. Overall, our findings indicated elevated levels of collagen and coagulation markers in the serum of SCLC patients and identified biomarkers that were responsive to therapy.

## METHODS

### Patient samples

#### Study design and patient demographics

This observational study included the medical records and blood samples of patients with SCLC, diagnosed and treated with chemotherapy at the Department of Oncology, Aalborg Hospital, Denmark, between March 2015 and September 2017. Pedersen et al. previously described the inclusion and exclusion criteria for the study.^23^ In brief, all patients followed the Danish standard treatment guidelines for chemotherapy involving platinum and a topoisomerase inhibitor. Additionally, a confirmed diagnosis of SCLC through histopathological and/or cytological analysis was mandatory for all patients included in the study. The exclusion criteria of this study encompassed several conditions, including a history of systemic chemotherapy for lung cancer, ongoing anticoagulation therapy (although platelet inhibitors, acetylsalicylic acid, and clopidogrel were allowed), a significant risk of or ongoing active bleeding, severe coagulopathy (such as hemophilia) or liver dysfunction, recent intracranial hemorrhage or central nervous system surgery within the past 3 months, treatment with any experimental agents, or participation in other clinical trials. Upon initial diagnosis, relevant clinical information, medication administration details, treatment information, radiological evaluations, and routine laboratory test results were gathered and recorded as baseline data. The staging of SCLC was determined using the seventh edition of the tumor, lymph node, and metastasis (TNM) classification for lung cancer.^24^ The North Denmark Region Committee on Health Research Ethics approved this study (N‐20140055), which was reported to the Danish Data Protection Authority (2021‐000206). The study adhered to the principles outlined in the Declaration of Helsinki. Prior to enrollment, all participants provided written informed consent. For comparative purposes, blood samples were obtained from 11 healthy controls with similar age and gender proportions from BioIVT (Table 1). According to the vendor, sample collection was approved by an Institutional Review Board or Independent Ethical Committee, and volunteers gave their informed consent. (Western Institutional Review Board, Inc. WIRB®Protocol #20161665). All investigations were conducted according to the Helsinki declaration.

**TABLE 1:** Clinicopathological characteristics of the cohort.

Characteristic	Healthy controls *N* = 11	SCLC *N* = 16	*p*‐value^a^
Gender, *n* (%)			>0.99
F	5 (45)	7 (44)	
M	6 (55)	9 (56)	
Age, median (range)	55 (41–72)	66 (51–76)	0.063
Smoking Y/N, *n* (%)			
Y	‐	15 (94)	
Unknown	11 (100)	1 (6)	
Cancer stage, *n* (%)			
IIIA	‐	3 (19)	
IIIB	‐	1 (6)	
IV	‐	12 (75)	
Treatment regimen, *n* (%)			
Carbo/Etopo	‐	16 (100)	

^a^ Fisher's exact test; Wilcoxon rank sum test.

#### Blood collection and sample preparation

Blood samples were collected from SCLC patients at the time of enrollment (baseline samples) and before initiating the third chemotherapy cycle (treated samples). Blood was collected from the median cubital vein using a 21‐gauge needle in 10 mL clot activator tubes (BD Vacutainer). After sample collection, the blood was centrifuged twice at 2500 × *g* for 15 min at room temperature to obtain serum. After each centrifugation step, serum samples were aspirated to approximately 1 cm above the buffy coat or pellet. Finally, serum samples were aliquoted, snap‐frozen using liquid nitrogen, and stored at −80°C until further analyses.

#### ELISA protocols

A panel of immunoassays that use monoclonal antibodies raised against epitopes in ECM proteins was used to quantify their concentrations in circulation (Table 2). The biomarkers are listed in the table below with reference to the originally published details describing the technical validation of the assays. Assays were conducted in accordance with the manufacturer's instructions (Nordic Bioscience A/S). All analytes were measured in duplicates and reported as the mean value per sample. The intra‐ and inter‐assay variances were validated for all assays to be <10% and < 15%, respectively. If the measured biomarker levels in a sample were below the lower limit of quantification (LLOQ), as determined in the validation of each assay, the level for that sample was changed to be equal to the LLOQ value.

**TABLE 2:** The biomarkers included in the study.

Number	Biomarker name	Epitope description	Related to biological process	Reference
1	PRO‐C1	A fragment of N‐terminal type I collagen	Bone formation, ECM formation	25
2	C1M	A fragment of type I collagen released by MMP	Inflammation‐driven degradation, ECM degradation	26
3	PRO‐C3	A fragment of N‐terminal type III collagen	Fibrogenesis, ECM formation	27
4	PRO‐C11	A fragment of N‐terminal type XI collagen	Fibrogenesis, ECM formation	28
5	PRO‐C19	A fragment of C‐terminal type XIX collagen	ECM remodeling	16
6	PRO‐C22	C‐terminal of type XXII collagen, neoepitope specific	Connective tissue formation	29
7	PRO‐FIB	Neo‐epitope of thrombin‐mediated degradation of fibrinogen	Clot formation	30
8	vWF‐N	Released N‐terminal propeptide of von Willebrand factor	von Willebrand factor activation	31

*Note*: 1–5: ECM biomarkers. 7–8: Wound healing biomarkers.

*Source*: https://www.nordicbioscience.com/products/biomarkers.

#### Statistical analysis

Differences in gender distribution between the patients with SCLC and the healthy individuals were evaluated using Fisher's exact test. Differences in age distribution were evaluated using the Wilcoxon rank sum test. Biomarker levels were log‐transformed before any formal testing. Differences in biomarker levels between the patients with SCLC at baseline and the healthy individuals were evaluated using *t*‐tests. The difference in biomarker levels between cancer stages (stage III and IV) was evaluated using *t*‐tests. The correlation between levels of different biomarkers was evaluated using Pearson correlations, and *p*‐values were adjusted using the Holm adjustment. Biomarker discrimination between patients with SCLC at baseline and healthy individuals was evaluated using receiver operating characteristics (ROC) curves summarized by the area under the curve (AUC) as well as the sensitivity, specificity, positive predictive value (PPV), and negative predictive value (NPV) at the biomarker cutoff where the Youden index was maximized. The difference in biomarker levels before and after treatment was evaluated using paired *t*‐tests and subsequently summarized into a heatmap using fold‐change in biomarker levels relative to baseline and clustered using “Euclidean” distances and the “ward.D2” method of the “ComplexHeatmap” R‐package.^32^ In general, a *p*‐value below 0.05 was considered statistically significant. Asterisks indicate the following significance levels: *p < 0.05; **p < 0.01; ***p < 0.001; and ****p < 0.0001. Statistical analysis and graphs were compiled in R version 4.2.2 R Core Team (2022).

## RESULTS

To determine the presence of circulatory ECM and wound healing peptides in SCLC patients, we quantified a range of biomarkers using ELISAs. We compared the average serum levels of these biomarkers at baseline (BL) and after chemotherapy treatment (TR) to those of healthy individuals. The biomarkers included collagen formation markers such as PRO‐C1 (internal epitope on the N‐terminal propeptide of type I collagen, also known as PINP), PRO‐C3 (ADAMTS‐2 generated cleavage of the N‐terminal propeptide of type III collagen) and PRO‐C11 (released N‐terminal propeptide of type XI collagen) as well as collagen degradation markers such as C1M, a marker of matrix metalloproteinase (MMP)‐mediated degradation of type I collagen, and PRO‐C19 and PRO‐C22 targeting the C‐terminus of type XIX and type XXII collagen, respectively. Furthermore, two wound healing markers were incorporated: PRO‐FIB (reflecting thrombin‐mediated degradation of fibrinogen) and vWF‐N (released N‐terminal pro‐peptide of vWF).

### Biomarkers elevated in advanced SCLC compared to healthy controls

**FIGURE 1:**
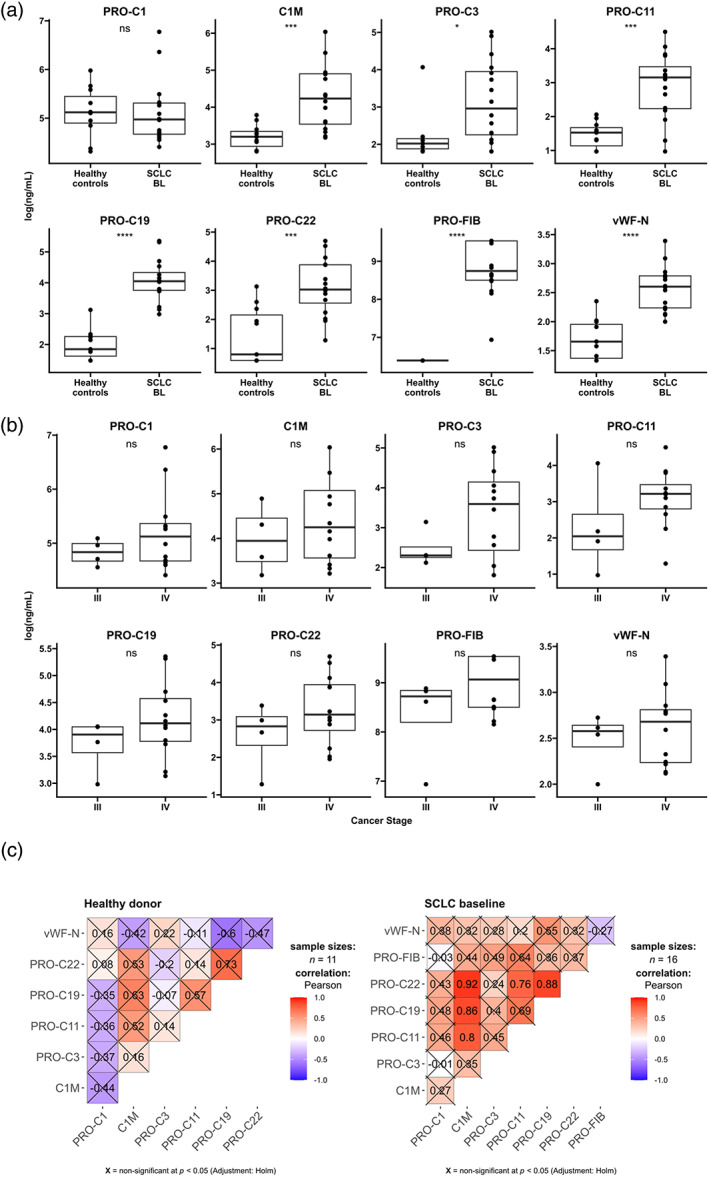
(a) Log‐transformed biomarker levels in healthy controls (*n* = 11) and baseline (BL) small cell lung cancer (SCLC) serum samples (*n* = 16). Biomarker levels are presented as Tukey‐style boxplots with data points. Samples measuring below the quantification limit were given the value of that limit, as determined in the validation of each assay. A *t*‐test was used to evaluate differences in biomarker levels. (b) Log‐transformed biomarker levels in baseline SCLC serum samples of stage III (*n* = 4) and stage IV (*n* = 12). (c) Correlation matrix of the biomarkers in healthy controls (left, *n* = 11) and baseline SCLC serum samples (right, *n* = 16). Value and color correspond to Pearson correlation coefficients and *p*‐values were adjusted using the Holm adjustment. PRO‐FIB was not included in the correlation matrix of healthy donors because all measurements were below the quantification limit.

For all biomarkers, except for PRO‐C1, significantly elevated mean levels in baseline SCLC samples compared to healthy controls (Figure 1a) were observed. PRO‐FIB exhibited the largest fold‐change, with a 14‐fold mean increase in biomarker levels compared to healthy controls. This was followed by a nine‐fold increase in PRO‐C19. The remaining biomarkers displayed fold changes ranging from 1.2 to six‐fold increases. Overall, the serum biomarker levels in SCLC demonstrated considerable variation within the group, suggesting that the biomarkers possessed an adequate dynamic range to identify differences in the serum of SCLC patients. Notably, none of the PRO‐FIB measurements in the serum of healthy controls were above the limit of quantification. In addition, all biomarkers were generally elevated in stage IV SCLC compared to stage III SCLC, but none of the differences were statistically significant (Figure 1b). Similarly, no significant associations were found between biomarker levels and the gender or age of individuals (Figures S1 and S2). In baseline SCLC samples C1M, PRO‐C11, PRO‐C19, and PRO‐C22 appeared to share strong correlations. The other combinations of markers shared moderate correlations, but none reached statistical significance. Similarly, no correlations reached statistical significance in the healthy donors (Figure 1c). In summary, higher levels of circulating ECM fragments were detected in SCLC patients compared to healthy individuals and some of the biomarkers correlated.

### Diagnostic accuracy

In addition to examining elevated levels of the biomarkers, we assessed the ability of each biomarker to distinguish between healthy and SCLC levels as a measure of baseline diagnostic accuracy. At baseline, all markers except for PRO‐C1 could effectively discriminate between healthy and SCLC patients (Table S3). PRO‐C19 and PRO‐FIB performed well, with AUC values of 0.99 and 1.00, respectively. Sensitivity, specificity, PPV, and NPV were also excellent for PRO‐FIB and PRO‐C19, with PRO‐C19 misclassifying only one individual. vWF‐N, PRO‐C22, PRO‐C11, C1M, and PRO‐C3 exhibited good classification performance with AUC values ranging from 0.8 to 0.96. This collectively indicates a potential diagnostic significance for these biomarkers.

### Biomarker changes after chemotherapy

**FIGURE 2:**
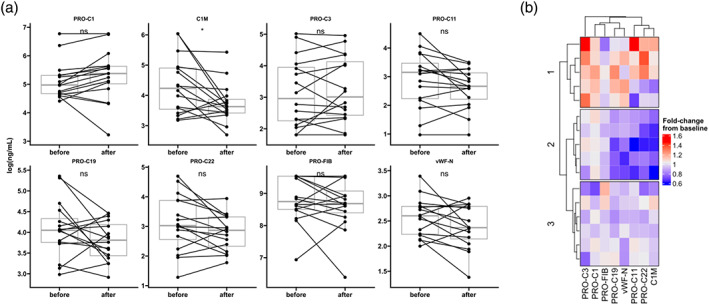
(a) Log‐transformed biomarker levels in small cell lung cancer (SCLC) serum samples at baseline (before) and follow‐up after chemotherapy (after). Biomarker levels are presented as Tukey‐style boxplots with data points and lines connecting paired baseline and follow‐up measurements. Paired *t*‐tests evaluated differences in biomarker levels. (b) Heatmap summarizing the fold‐change in biomarker levels at follow‐up relative to baseline. Hierarchical clustering was performed using the Euclidean distance metric and the Ward.D2 clustering method.

To assess whether any of the biomarkers changed after chemotherapy, we compared biomarker levels pre‐ and post‐treatment, which uncovered a significant decline in C1M levels (Figure 2a, Table S4). Although the remaining markers did not show significant changes, PRO‐C1 tended to increase after treatment, PRO‐C3 remained unchanged, and the rest of the biomarkers generally decreased after treatment but without reaching statistical significance (Table S4). The observed increase in PRO‐C1 (a marker of type I collagen formation) and a decrease in C1M (a marker of type I collagen degradation) might suggest that a net increase in type I collagen could occur after chemotherapy. However, in the patients where C1M decreased the most (denoted subgroup 2 in Figure 2b), PRO‐C1 did not change much. Instead, in subgroup 1, both C1M and PRO‐C1 seemed to increase. For all biomarkers, three distinct subgroups emerge (Figure 2b): subgroup 1, characterized by increased levels of most biomarkers, excluding PRO‐FIB, suggesting heightened ECM turnover in these patients; subgroup 2, where the majority of biomarkers, except for PRO‐C1, tend to decrease; and subgroup 3, in which the alterations are less evident, and no definitive patterns can be identified. Overall, these findings indicate that this panel of biomarkers may be modulated by chemotherapy and can potentially identify subgroups of patients based on the changes in their ECM profile as the disease advances and treatment progresses.

## DISCUSSION

In this preliminary study, we investigated serum ECM and wound healing biomarkers, an area not previously explored in small cell lung cancer. We examined serum biomarkers from 16 patients with small cell lung cancer before and after chemotherapy, along with 11 healthy controls. We found elevated amounts of collagen type I degradation, collagen type III and XI formation, and collagen types XIX and XXII. Additionally, we found high levels of fibrin and von Willebrand factor A production. There was a decrease in type I collagen degradation observed after chemotherapy. In general, we detected increased levels of collagen and wound‐healing markers in the serum of SCLC patients, highlighting the potential of noninvasive quantification of ECM proteins as biomarkers in SCLC.

To capture the most relevant collagen biology for SCLC, we included fibrogenic or collagen formation markers (PRO‐C1, PRO‐C3, and PRO‐C11) and fibrolysis or collagen degradation markers (C1M). Recent studies have also revealed that the minor collagens, such as the fibril‐associated collagens, are deregulated in cancer (PRO‐C19 and PRO‐C22).^16^, ^29^ In addition, although the collagen formation markers are closely associated with a fibrotic phenotype, the collagen degradation marker C1M is driven by inflammation.^26^ Importantly, the collagen family is large and diverse, spanning 28 different proteins. Therefore, it may be that other collagens not included in the current study may prove useful in SCLC as biomarkers.

A significant challenge in SCLC biomarker research is the difficulty in acquiring tissue samples, as surgical procedures are uncommon, and SCLC tumors tend to be small and difficult to biopsy.^10^ The utilization of blood‐based biomarkers is a viable solution to this issue. Blood collection is more accessible and can be performed at various convenient times. Skeptics may contend that blood is too intricate of a sample material and that the analyte concentration is too low to measure accurately, thereby rendering it difficult to deduce any differences among patients. However, our study has shown the potential of using blood‐based biomarkers in SCLC, where we quantified our biomarkers in the serum of patients with SCLC in quantities high enough to capture patient‐to‐patient variation in biomarkers. This opens up avenues for future research to build upon and broaden our understanding in this field.

Other examples of serum markers,^33^ as well as circulating tumor cells^34^ and tumor mutational burden,^35^ appear promising, but to date, these biomarkers have shown limited clinical utility and have yet to mature to regular use in the clinic. Therefore, there is still a pressing need to develop novel biomarkers in the SCLC space. Although chemotherapy is used to treat most SCLC patients, there is a lack of biomarkers that predict chemotherapy efficacy and a lack of biomarkers reflecting the development of chemoresistance. Our results of C1M being modulated by chemotherapy point to some potential in this context, but it remains unclear what causes the change in C1M. Is the observed change indicative of treatment effectiveness, or does it instead suggest treatment failure and the emergence of resistance? Resistance to chemotherapy is a common phenomenon in SCLC. Excessive production of ECM proteins activates signaling in SCLC cells through β1 integrin to prevent etoposide‐induced apoptosis.^17^, ^34^, ^36^ Positive β1 integrin staining in SCLC tissue biopsies is also associated with poor survival.^37^ A recent study also demonstrated that a subset of chemotherapy resistant SCLC cells had more mesenchymal EMT scores.^38^ Interestingly, we observed a significant decrease in the collagen degradation marker C1M, potentially leading to a net increase in type I collagen, possibly related to acquired treatment resistance. The current study is not designed to assess this potential, but future research is warranted.

The interaction between the tumor microenvironment (TME) and tumor cells is continuous, as it provides the signals for tumor growth and survival. One of the main parts of the TME is cancer‐associated fibroblasts (CAFs). Several studies have shown that CAFs release a variety of substances such as growth factors, cytokines, and enzymes that enhance the capacity of cancer cells to promote stemness, promote angiogenesis, modify the ECM, evade the immune system, and develop resistance to treatment.^39^ Tumor cells reciprocally can initiate and sustain CAF activation to establish a feed‐forward cycle.^39^ Despite these observations, the ECM remains underexplored in the SCLC field. Our research group has previously demonstrated that fragments of ECM proteins can be quantified reliably in blood, which provides value as cancer biomarkers.^40^, ^41^, ^42^ In the current study, we demonstrate that this also extends to SCLC. It may therefore be speculated, as explored in previous studies, that these fragments of ECM proteins may be markers of CAF activity. In addition to being measures of CAF activity, the fragments we measure may result from other cell activities, including macrophages that secrete MMPs.

The interactions of tumor cells with CAFs promote EMT and metastasis. The mechanical properties and composition of the ECM play a key role in regulating EMT. Reciprocally, the process of EMT induces cellular changes that result in ECM remodeling creating a feedback loop that is tightly regulated in healthy tissues but is deregulated in cancer.^43^ This cell interaction has recently been demonstrated in SCLC using a lung fibroblast coculture model.^8^ Therefore, the consequences of ECM remodeling extend to the SCLC cells. The biomarkers we measure in the current study may indicate a broader network of signaling alterations that involve fibroblasts and SCLC cells. Furthermore, EMT cell states are dynamic and can be reversed,^8^ so a novel mode of action to treat SCLC could involve normalization of the ECM to temper protumor signaling in both fibroblasts and SCLC cells. In this context, the biomarkers identified in the current study may also be valuable.

One of the genes altered most often in SCLC is COL11A1, the gene coding for type XI collagen.^44^, ^45^ Our panel of immunoassays also included an epitope on the propeptide of type XI collagen (PRO‐C11). The release of this epitope to the circulation indicates type XI collagen formation. Consistent with a role in SCLC, we observed elevated levels of PRO‐C11. COL11A1 is also associated with chemotherapy resistance in NSCLC^46^ and ovarian cancer^47^ and is induced by cisplatin treatment in NSCLC cells.^46^ Nonetheless, our current study did not support any modulation of PRO‐C11 following chemotherapy, which could indicate a disparity between NSCLC and SCLC in this aspect.

Patients with SCLC are more susceptible to thrombogenic events, and coagulation factors have previously been associated with SCLC.^23^, ^48^ High levels of pretreatment D‐dimer or fibrinogen have been linked to inferior survival outcomes^49^, ^50^, ^51^ and poorer response to chemotherapy.^51^, ^52^ In the current study, we included two blood coagulation markers: the PRO‐FIB marker, which reflects the thrombin‐mediated degradation of fibrinogen to fibrin, and vWF‐N, which reflects the formation of vWF. Both markers were elevated in SCLC serum compared to controls, although none were modulated by chemotherapy. It could be speculated that this increase reflects an acute phase reaction, where, in response to inflammation, both fibrinogen and von Willebrand factor would be expected to increase. It remains to be seen how these biomarkers are associated with patient outcomes and cancer‐associated thromboembolism.

The current study was a preliminary investigation and had several limitations. The small sample size limits the strength of the analysis and necessitates a follow‐up study to confirm the results of the current study. In addition, the clinical information available for patients with SCLC was limited. Their response to therapy and survival outcome was unavailable, limiting the current study to comparing biomarker levels with no regard for clinical outcome. Another important limitation is that the source tissue from which the biomarkers are released into circulation is not known. This, combined with the fact that the included biomarkers may also be elevated in other conditions, limits what we can infer about the underlying biology. We can infer that some activity is elevated in SCLC, but exactly how this is connected to SCLC is not known. Finally, given the entirely exploratory nature of our current study, it is essential to conduct further research that can replicate and expand upon our findings.

In conclusion, we investigated biomarkers of ECM proteins in the serum of 11 healthy individuals and 16 patients with small cell lung cancer before and after chemotherapy. We found increased levels of collagen type I degradation, type III, and type XI formation and increased levels of type XIX, type XXII collagen, and von Willebrand factor A and fibrin formation. These findings suggest that ECM and blood clotting biomarkers may have biomarker potential in SCLC. Additionally, we observed a decrease in the degradation of type I collagen after chemotherapy, which could indicate treatment response or acquired chemoresistance. These preliminary findings warrant further studies to explore the use of ECM proteins as potential biomarkers in SCLC.

## AUTHOR CONTRIBUTIONS

Jeppe Thorlacius‐Ussing: Conceptualization; methodology; formal analysis; investigation; resources; writing–original draft; writing–review and editing; visualization; project administration. Morten Asser Karsdal: Supervision; funding acquisition; writing–review and editing. Nicholas Willumsen: Conceptualization; supervision; writing–review and editing. Søren Risom Kristensen: Resources; writing–review and editing. Shona Pedersen: Conceptualization; resources; writing–review and editing; project administration.

## CONFLICT OF INTEREST STATEMENT

Jeppe Thorlacius‐Ussing, Morten Asser Karsdal, and Nicholas Willumsen are employed at Nordic Bioscience A/S, a company developing serological biomarkers. Morten Asser Karsdal and Nicholas Willumsen own stock in Nordic Bioscience A/S.

## Supporting information


**Data S1.** Supporting Information.
