# Supplementary material for: Preliminary investigation of elevated collagen and blood‐clotting markers as potential noninvasive biomarkers for small cell lung cancer
Source: Thorac Cancer. 2023 Aug 19;14(28):2830–8. doi: 10.1111/1759-7714.15066 (PMC10542464; doi:10.1111/1759-7714.15066)
Supplement: Supplementary file 1 — Data S1. Supporting Information. [file TCA-14-2830-s001.docx]

# Supplementary


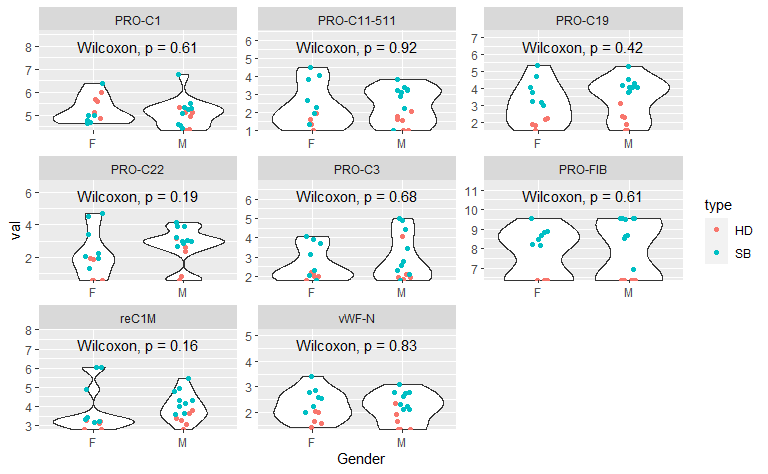


Figure S 1


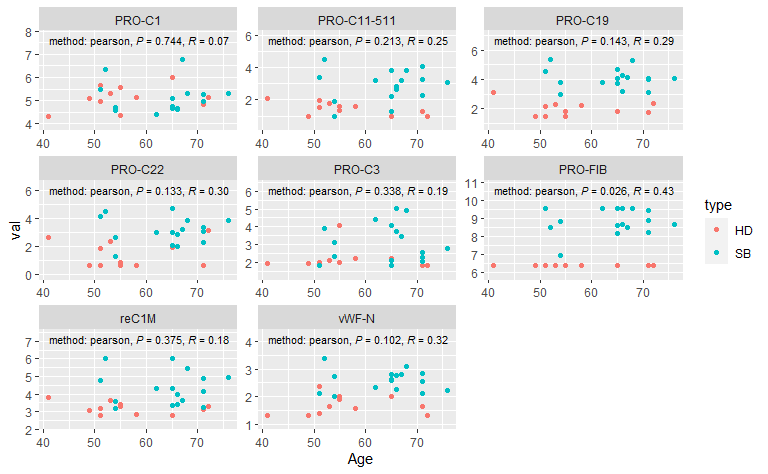


Figure S 2

| **Biomarker** | **AUC** | **Cutoff (ng/mL)** | **Youden Index** | **Sensitivity** | **Specificity** | **PPV** | **NPV** |
| --- | --- | --- | --- | --- | --- | --- | --- |
| PRO-FIB | 1 | 1,028.8 | 1 | 1 | 1 | 1 | 1 |
| PRO-C19 | 0.99 | 22.97 | 0.94 | 0.94 | 1 | 1 | 0.92 |
| vWF-N | 0.96 | 8.31 | 0.85 | 0.94 | 0.91 | 0.94 | 0.91 |
| PRO-C22 | 0.89 | 7.06 | 0.66 | 0.94 | 0.73 | 0.83 | 0.89 |
| PRO-C11 | 0.89 | 8.85 | 0.81 | 0.81 | 1 | 1 | 0.79 |
| C1M | 0.89 | 30.31 | 0.63 | 0.81 | 0.82 | 0.87 | 0.75 |
| PRO-C3 | 0.8 | 9.95 | 0.66 | 0.75 | 0.91 | 0.92 | 0.71 |
| PRO-C1 | 0.56 | 164.67 | 0.26 | 0.64 | 0.62 | 0.54 | 0.71 |

Table S 3. The diagnostic accuracy of the biomarkers using a ROC-curve analysis.
Abbreviations: Area under the curve (AUC), positive predictive value (PPV), negative predictive value (NPV)

| **Biomarker** | **Fold-change** | **p-value** |
| --- | --- | --- |
| PRO-C1 | 1.301 | 0.177 ns |
| C1M | 0.448 | 0.019 * |
| PRO-C3 | 1.033 | 0.518 ns |
| PRO-C11 | 0.613 | 0.066 ns |
| PRO-C19 | 0.668 | 0.130 ns |
| PRO-C22 | 0.576 | 0.064 ns |
| PRO-FIB | 0.872 | 0.360 ns |
| vWF-N | 0.842 | 0.088 ns |

Table S 4: Summary of the mean fold-change between baseline and after chemotherapy for SCLC patients and the p-value for the paried t-test.
